# Pockets of progress amidst persistent racial disparities in low birthweight rates

**DOI:** 10.1371/journal.pone.0201658

**Published:** 2018-07-31

**Authors:** Samantha S. Goldfarb, Kelsey Houser, Brittny A. Wells, Joedrecka S. Brown Speights, Les Beitsch, George Rust

**Affiliations:** 1 Department of Behavioral Sciences and Social Medicine, College of Medicine, Florida State University, Tallahassee, FL, United States of America; 2 Department of Health Sciences, College of Health Professions and Sciences, University of Central Florida, Orlando, FL, United States of America; 3 Department of Family Medicine and Rural Health, College of Medicine, Florida State University, Tallahassee, FL, United States of America; 4 Center for Medicine and Public Health, College of Medicine, Florida State University, Tallahassee, FL, United States of America; University of Zurich, SWITZERLAND

## Abstract

Racial disparities persist in adverse perinatal outcomes such as preterm birth, low birthweight (LBW), and infant mortality across the U.S. Although pervasive, these disparities are not universal. Some communities have experienced significant improvements in black (or African American) birth outcomes, both in absolute rates and in rate ratios relative to whites. This study assessed county-level progress on trends in black and white LBW rates as an indicator of progress toward more equal birth outcomes for black infants. County-level LBW data were obtained from the 2003 to 2013 U.S. Natality files. Black LBW rates, black-white rate ratios and percent differences over time were calculated. Trend lines were first assessed for significant differences in slope (i.e., converging, diverging, or parallel trend lines). For counties with parallel trend lines, intercepts were tested for statistically significant differences (sustained equality vs. persistent disparities). To assess progress, black LBW rates were compared to white LBW rates, and the trend lines were tested for significant decline. Each county’s progress toward black-white equality was ultimately categorized into five possible trend patterns (n = 408): (1) converging LBW rates with reductions in the black LBW rate (decreasing disparities, n = 4, 1%); (2) converging LBW rates due to worsening white LBW rates (n = 5, 1%); (3) diverging LBW rates (increasing disparities, n = 9, 2%); (4) parallel LBW rates (persistent disparities, n = 373, 91%); and (5) overlapping trend lines (sustained equality, n = 18, 4%). Only four counties demonstrated improvement toward equality with decreasing black LBW rates. There is significant county-level variation in progress toward racial equality in adverse birth outcomes such as low birthweight. Still, some communities are demonstrating that more equitable outcomes are possible. Further research is needed in these positive exemplar communities to identify what works in accelerating progress toward more equal birth outcomes.

## Introduction

Worldwide, low birth weight contributes to 60–80% of all neonatal deaths [1] and is associated with a myriad of adverse outcomes, ranging from neonatal complications [2] to developmental delays [3] to sudden infant death syndrome [4]. Unfortunately, low birthweight (LBW) rates continue to be almost twice as high among African American (hereafter, black) infants compared to white infants (13.35% vs. 6.93%) [5].

One of the strongest predictors of infant mortality is LBW, a category which can include both premature babies and those who are small for gestational age. Infants who are LBW weigh 2,500 grams (5.5 pounds) or less at birth, while very low birthweight (VLBW) infants weigh 1,500 grams (3.25 pounds) or less at birth [6]. VLBW infants in particular are up to 110 times more likely to die within the first year of life than their normal birthweight counterparts [7], but the risks associated with VLBW are not a threshold phenomenon, and there is a continuum of risk across all levels of low birthweight for both immediate and long-term health and developmental issues [6]. Although there has been an 18% decline in IMR among black women from 2005–2013 [8], the VLBW rates have not made significant improvements over the past two decades [5]. The impact on racial infant mortality disparities is substantial, with VLBW accounting for nearly two-thirds of the black-white gap in IMR in the U.S. [9,10].

While much of the literature on perinatal outcomes reinforces the theme that racial disparities are pervasive and persistent, Fry-Johnson and colleagues have demonstrated that disparities in infant mortality are not inevitable and that there are counties with more equitable birth outcomes [11]. Recent studies have emphasized the impact of “place” on health outcomes, with local-level variation demonstrating both positive and negative deviance [12]. Kershenbaum et al demonstrated the wide variation that exists in premature birth rates across U.S. counties, and the tremendous variety of social and environmental “determinants” which combine in complex ways to explain this variation [13]. Similarly, Brown Speights and colleagues showed substantial state-level variation in progress toward the elimination of racial disparities in infant mortality [14]. Rossen et al described significant geographic variation in county-level infant mortality disparities as measured by both absolute rate differences and relative rate ratios [12]. This recent research suggests that there may be positive exemplars of counties demonstrating progress toward racial equality in health outcomes, which could inform new approaches to move communities from high-disparity patterns to optimal and equitable health outcomes.

Therefore, we conducted this study to assess trends in county-level black-white disparities in LBW rates, in order to identify counties with trend lines demonstrating progress toward more equitable outcomes. Positive progress at the county-level would be demonstrated by improvement in outcomes (i.e., decreasing rate of LBW births for both racial groups, especially black babies) with concomitant improvement in relative disparities (i.e., decreasing black-white rate ratios as demonstrated by black and white LBW rate trend lines converging toward more equal outcomes). Such positive progress in even a small proportion of counties would demonstrate the real possibility of eliminating racial disparities in LBW rates and achieving both optimal and equitable outcomes for all babies, regardless of race.

## Materials and methods

County-level LBW data were obtained from the 2003 to 2013 U.S. natality files. Data were obtained with approval from the National Association for Public Health Statistics and Information Systems through the all-county data files for counties with populations of 100,000 or greater. After selecting counties with at least 10 LBW babies in each racial group by year to protect confidentiality, 408 counties from 50 states and the District of Columbia were eligible for study inclusion, with data restricted to live births for whom race was categorized as non-Hispanic white or black. While this number represents only 13% of US counties, these counties include approximately 85% of all the black births in this country on average for each year included in the analysis. Data were deidentified before receipt by the authors, and this study was approved by the Florida State University Institutional Review Board.

In order to determine how disparities are changing over time, we modeled separately for each year the county-level incidence rate of LBW babies for all black births and all white births. We chose to model incidence rates as opposed to rate ratios because, while rate ratios can tell you *when* a county’s rates are converging, they cannot tell you *how* a county’s rates are converging. That is, they cannot distinguish between whether a county’s rates are converging because the black rate is declining or because the white rate is increasing.

### Racial disparity trend pattern categorizations

Based on the work of Rust et al [15,16], we initially categorized each county into 1 of 4 black-white LBW racial disparity trend patterns. These categories were:

1) Persistent disparities (black-white LBW rates roughly parallel but non-overlapping);
2) Sustained equality (black-white LBW rates essentially equal–trend lines overlapping throughout study period);
3) Convergent (black-white LBW rates converging towards equality in a statistically significant manner); and
4) Divergent (black-white LBW rates diverging to greater disparities in a statistically significant manner).

### Analysis

Each county’s black LBW rate and white LBW rate from 2003–2013 were calculated annually. These rates determined if, and to what extent, counties made progress towards equal (black LBW declining and converging towards equality) levels of LBW rates.

We conducted a log-linear model using OLS regression with race represented as a dummy variable for black rates, year included as a linear trend, and with each county modeled separately via a full set of county-specific dummies and corresponding county-specific interaction terms. This specification estimated the trend patterns for all counties by race across the 11-year study period. For this model, the county-specific time by race interaction served as the main independent variable, and the log of the low birthweight rate for each race was the dependent variable. The analysts generated the code for the analysis using SAS software, version 9.4. Based on the model results, counties were then categorized by trend pattern. A convergent trend pattern was identified when the estimated slope for the black rate was statistically significantly negative, the estimated slope for the white rate was statistically flat, and the difference between the two rates (the coefficient on the interaction term) was statistically significantly negative. A divergent trend pattern was identified when the estimated slope for the black rate was flat or statistically significantly positive, the estimated slope for the white rate was statistically significantly negative or flat, and the difference between the two rates was statistically significantly positive. Sustained equality and persistent disparity were identified when the difference between the two rates was statistically indistinguishable, with sustained equality determined when the difference between the intercepts was not statistically significant and persistent disparity determined when the difference in intercepts was statistically significant and positive for the black rate. Recent discussions on multiple hypothesis testing have emphasized the need to adjust *p*-value thresholds to reduce the probability of a Type I error. Accordingly, all hypothesis tests were two-tailed and conducted at the 0.005 level [17].

After these classifications were made, more detailed information (population size, percent changes in black-white LBW rates and rate ratios) was compiled for counties demonstrating positive progress with convergent trend patterns and concomitant reductions in black LBW rates. A graph of aggregated data for all counties in this trend pattern category based on the black and white LBW rates from 2003 to 2013 was also produced. This graph provides a visual representation of the convergence trend to better understand the counties that are achieving progress towards equality in black and white LBW rates.

We also projected the total potential number of low birthweight births averted if we had eliminated the gap in black-white LBW rates in all included counties during the study period. This was conducted by setting the white LBW rate as a function of the black total births and LBW births per year per county for a cumulative total. That is, for each year and county, the following calculation was made: Potential averted black LBW births = Total number black LBW births–(white LBW rate/100 * Total number of black births).

## Results

Table 1 depicts U.S. counties categorized according to their racial disparity trend pattern. Overall, 1% of counties demonstrated black-white convergence with reductions in black LBW rates, 1% also demonstrated convergence primarily due to worsening white LBW rates over time, 2% were divergent, 4% demonstrated sustained equality, and 91% were persistently unequal. Therefore, the most common trend pattern was parallel and persistently unequal black and white trend lines. Those counties with sustained equality mostly demonstrated persistent gaps between black and white LBW rates, but their trend lines were not statistically significant different. However, one exemplar county that stood out in this category was Middlesex County, Massachusetts, which demonstrated low LBW rates among both black and white infants throughout the entire study period (mean 5.7% and 4.6%, respectively).

**Table 1:** U.S. Counties categorized by black/white racial disparity patterns for low birthweight from 2003 to 2013 (N = 408).

Region	Description	# of Counties	% of Total
Convergence with reductions in black LBW and no increase in white LBW rate	Rates converging due to decrease in BLBW rates and flat WLBW rates	4	1%
Convergence due to increase in white LBW rate	Rates converging due to 1) decreasing BLBW rates *and* increasing WLBW rates OR 2) flat BLBW rates and increasing WLBW rates	5	1%
Sustained Equality	BLBW and WLBW rates not statistically different over time	18	4%
Persistent Inequality	BLBW and WLBW rates remain statistically different over time (but may be concurrently increasing, flat, or decreasing)	373	91%
Divergence	Rates diverging due to 1) increasing/flat BLBW rates while WLBW flat/decreasing	8	2%
**Total**		408	100%

BLBW = Black Low Birthweight

WLBW = White Low Birthweight

Table 2 depicts U.S. counties with convergent racial disparity trend lines with declining black LBW rates from 2003 to 2013. Overall, these converging counties have demonstrated substantial reductions in LBW disparities as measured by black-white rate ratios. These four counties include Craighead County, AR; Black Hawk County, IA; Delaware County, IN; and Stearns County, MN. Among those counties, percent changes in white LBW rates ranged from a 33% increase to an 8% decrease, while percent changes in black LBW rates ranged from a 10% to a 63% decrease. Nationally, the rates for black LBW declined slightly over time and remained unchanged for white LBW.

**Table 2:** U.S. counties with racial disparity trend lines converging with no increase in white LBW from 2003 to 2013.

County, State	Year	LBW, white	% Change*	LBW, black	% Change*	Black-White Rate Ratio	% Change^	Total Population
Craighead County, Arkansas	2003	4.2	33.3	10.5	-62.9	2.5	-120.0	84,953
	2013	5.6		3.9		0.7		101,678
Black Hawk County, Iowa	2003	5.0	-2.0	17.8	-57.9	3.6	-80.8	126,026
	2013	4.9		7.5		1.5		132,716
Delaware County, Indiana	2003	7.7	26.0	13.7	-9.5	1.8	-62.5	552,355
	2013	9.7		12.4		1.3		561,619
Stearns County, Minnesota	2003	7.8	-7.7	11.2	-15.2	1.4	-25.0	140,307
	2013	7.2		9.5		1.3		152,165
**National Average**	**2003**	**7.0**	0.0	**13.4**	-4.5	1.9	-11.1	**~290,100,000**
	**2013**	**7.0**		**12.8**		1.8		**~316,200,000**

*% change LBW rates calculated as (2013 rate– 2003 rate)/2003 rate * 100

^% change Rate Ratio calculated as (2013 rate ratio– 2003 rate ratio)/(2003 rate ratio − 1) * 100

**Fig 1:**
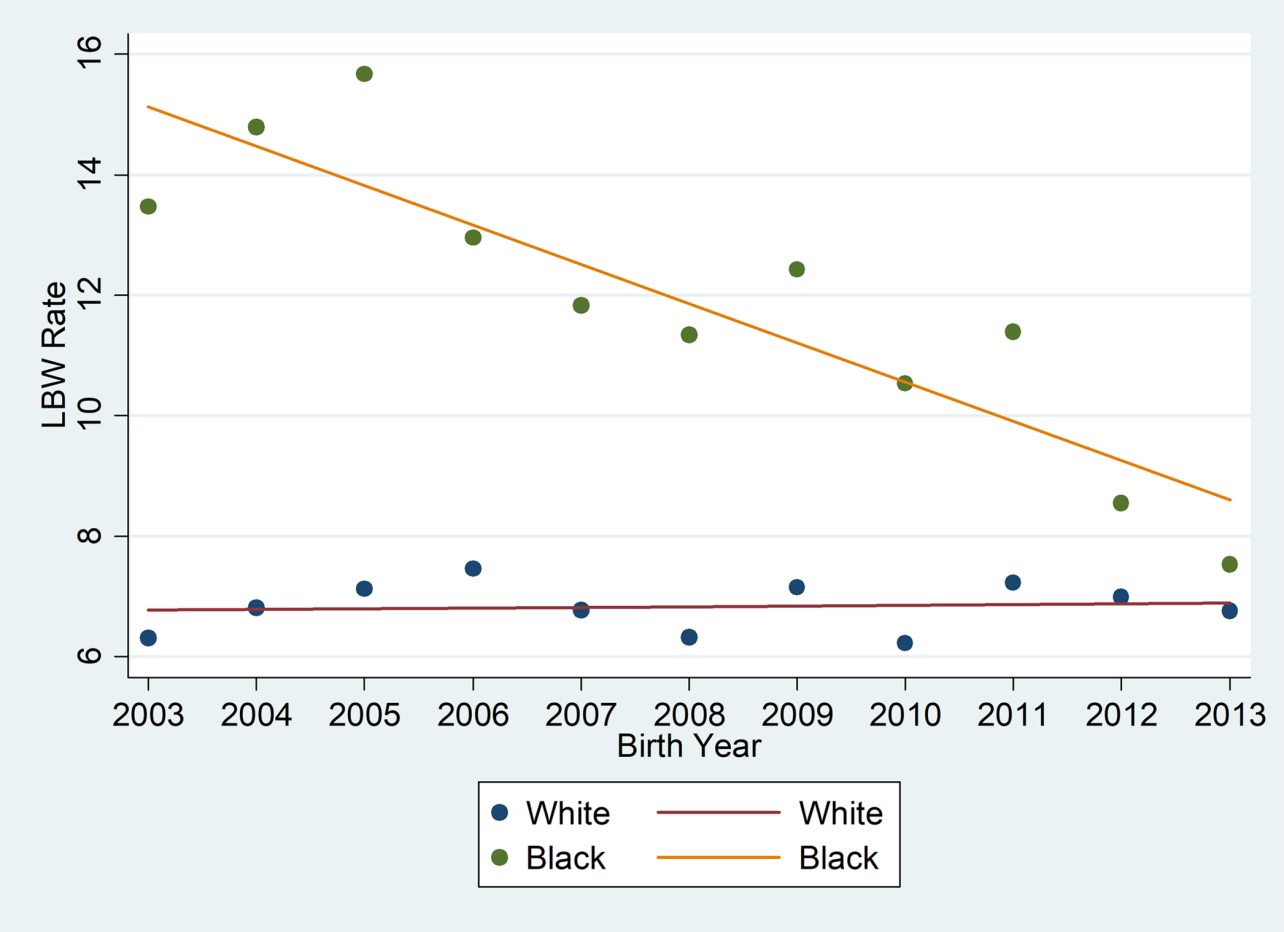
Aggregated LBW rates for U.S. counties with convergent racial disparity trend pattern (reductions in black LBW and no increase in white LBW) from 2003 to 2013.

Fig 1 depicts the aggregated LBW rate trend by race for counties in the convergent trend pattern identified above. As depicted in Fig 1, convergent counties overall demonstrate a reduction in black LBW rates over time, with little to no change in white LBW rates.

Additionally, the potential number of LBW births averted for all counties during the study period were black-white LBW rates to be equal was calculated to be 371,176. According to the most conservative estimate, an average LBW hospitalization stay in 2001 was estimated at $15,100. If we applied this to the study period, $5,604,757,600 in immediate hospitalization costs could have been avoided had black-white equality in LBW rates been achieved [18,19]. It is important to note that this estimate is extremely conservative as it does not adjust for inflation (estimated at $22,953.76 in 2012), it does not account for those LBW births that are VLBW (estimated in 2001 at $52,300-$65,600), and it does not account for the other direct and indirect costs associated with LBW births [18,19].

## Discussion

The purpose of this study was to better understand county-level black-white disparities in LBW rates, with the goal of identifying counties demonstrating progress toward equal outcomes. Our main finding is that not only is there significant U.S. county-level variation in both black and white LBW rates, but that there is also evidence of meaningful progress in specific counties.

The slow progress in achieving reductions in racial disparities in LBW rates has already been well documented at a national level, but the local-area variation provides a more nuanced picture. The four counties moving toward equitable outcomes reinforce the growing body of evidence that racial disparities in health outcomes are not inevitable [14,20]. From 2003 to 2013, these counties have demonstrated a converging pattern, moving from substantial racial disparities to closing the gap towards equality, with black LBW rates declining to meet white LBW rates. Convergence occurred in five additional counties because white LBW rates worsened, even as black LBW rates showed variable improvement. Additionally, 18 counties showed a pattern of sustained equality (overlapping black-white trend lines). The pattern of persistent disparities (parallel black and white trend lines) was most common (n = 373; 91%), although eight counties showed diverging trend lines (disparities worsening). These findings underscore the importance of measuring health equity by examining both relative disparities and absolute disparities as study outcomes.

We chose to examine all categories of LBW infants given their increased vulnerability for poor short- and long-term health outcomes, across a continuum of risk from extreme VLBW to mild LBW. While VLBW births are especially important given their correlation with infant mortality rates, especially in the black population, they are a subset of the larger problem of LBW births [19]. Interventions to decrease LBW birth broadly will directly impact the more severe adverse outcomes of VLBW and infant mortality [21].

Previous literature suggests that different risk factors and stressors may be associated with LBW outcomes in different racial groups. Black women who had a LBW infant were more likely to be of low socioeconomic status than white women, despite also being more likely to be in good health [22]. However, even among black and white women who receive adequate prenatal care and have a college education, black women historically experienced a LBW rate that was more than twofold higher than white women [23].

Across racial groups, neighborhood-level social determinants and individual psychosocial stressors can be difficult to tease apart as risk factors for LBW or very preterm birth. Data from the Early Childhood Longitudinal Study Birth Cohort linked to county-level neighborhood disadvantage index demonstrated that across all neighborhood strata, but especially within the most disadvantaged neighborhoods, LBW risk was exacerbated by the experience of stressful life events before conception [24]. El-Sayed et al found that socioeconomic position, maternal behaviors, demographics, and health access explained nearly a third of racial disparities in various causes of mortality in Michigan [25].

Chronic stress has been shown to be notably higher in the daily lived experiences of black women compared to whites [26,27], and stress perpetuated by racial discrimination can also have adverse biopsychosocial effects on both mother and infant [26]. A more sophisticated understanding of the interactive effects of racism on poor birth outcomes for black women over the life course also needs further consideration [28]. Studies in Newcastle, England showed that socioeconomic inequalities did not narrow over forty years (1961–2000), and indeed widened for preterm birth [29]. This is consistent with a review by Collins, et al and posited by Geronimus, regarding the effects of intergenerational minority status across the course of life for black women and the weathering impact of stress on maternal and child health outcomes [26,30]. Indeed, African American women experiencing interpersonal racism in 3 or more domains were 2.6 times more likely than white women to deliver a VLBW baby, even after controlling for other associated factors [31].

Although childhood poverty may have long-lasting and even epigenetic effects on women as they enter childbearing years, data on 2,463 women from 52 clinics in five Michigan communities suggests that upward mobility (moving from low-SES to middle or upper-class SES) could be significantly protective of fetal growth, as measured by the birth outcome of small for gestational age [32]. Upward economic mobility of black women is associated with decreased risk of preterm birth, unless the women were LBW themselves [30].

Structural equation modeling of preconception processes influencing birthweight using data from 1,580 mother-daughter pairs in National Longitudinal Survey of Youth found that birthweight was shaped not only by factors in women’s early background (especially education), but also by conditions dating back as much as three generations [33]. Two other studies have found race-specific intergenerational effects, with black mothers who themselves were born low birthweight or preterm being more likely to bear LBW infants, but white mothers born LBW or preterm had no increased risk of bearing an LBW infant [34,35].

Data on 3,869 women from the 1998–2000 Fragile Families and Child Wellbeing Study showed significant associations between known social and behavioral stressors (use of public assistance or public housing, smoking, drug use, and certain religious affiliations) and low birthweight, but did not fully explain the racial disparities in LBW [36]. Analysis of feto-infant mortality data (FIMR) in St. Louis from 2000 to 2009 found that VLBW contributed 50% of the excess FIMR countywide. Among those with VLBW, black maternal race was the strongest risk factor (40.6% of attributable risk), followed by inadequate prenatal care (19.8%) and gestational hypertension (12.0%). Medicaid coverage was protective against the adverse birth outcome of VLBW (population attributable risk = -14.5) [37].

Geographically granular analysis at the census tract and neighborhood levels in New York City found a positive association between residential segregation and low birthweight, after controlling for individual-level risk factors and neighborhood poverty [38]. Residential segregation also explained neighborhood variation in low birthweight rates and racial disparities across census tracts, which were not explained by neighborhood poverty alone [38].

Seeking to tease out the differential effects of individual and neighborhood-level factors, Vang and Elo examined the association between neighborhood minority diversity and infant birthweight, among black non-Hispanic women stratified by country of birth (U.S. born vs. foreign born). Black women born in Africa or the Caribbean had significantly higher birthweights than did U.S.-born black women, even after controlling for neighborhood deprivation, residential instability, individual-level sociodemographics, maternal health behaviors, and gestational age. Neighborhood non-white racial-ethnic diversity (minority heterogeneity) at the census tract level had a positive association with higher black infant birthweights, as did living in a majority-minority neighborhood [39]. Moving beyond risk factors and negative determinants, identifying communities with positive outcomes may help chart a path toward perinatal health equity. Kershenbaum et al found counties with paradoxically low preterm birth rates, even after controlling for social and environmental “determinants” [13]. Further research is needed to understand what enables these counties to achieve more optimal and equitable perinatal outcomes, and what explains the “residual effects” or unmeasured factors that make them outliers (positive deviants).

The trend of increasing LBW rates among white mothers in some counties also merits attention. Factors driving increases in white LBW rates in pockets of the country might include the impact of the economic downturn, increasing rates of opioid use and addiction, variable access to prenatal care, variation in smoking rates (including youth adoption of e-cigarettes), and other biopsychosocial stressors. Further study is needed to understand specific drivers of increasing white LBW rates in these counties.

Interventions to decrease prematurity and LBW births could be both obstetrical and social-contextual. For example, one survey at the hospital level demonstrated that LBW infants born in high-black (patient-mix) hospitals had more severe levels of nurse understaffing and higher rates of infection and infant discharge without breast milk than low-black hospitals [40]. Reducing elective deliveries and C-sections is also an important target of intervention at the provider and hospital level [41,42]. Structural factors, including state-level health policies, can also impact racial disparities in birth outcomes. For example, state funding levels for family planning and abortion have been found to be associated with reductions in infant mortality, even controlling for race and county-level covariates [43]. Smoking legislation, such as increasing cigarette taxes, can affect prenatal smoking, a major risk factor for low birthweight and adverse perinatal outcomes [44,45]. The establishment of a state Office of Minority Health has also been shown to impact racial disparities in birth outcomes, especially in states with low Medicaid funding levels [46].

Interventions to improve these birth outcomes, especially in minority communities, often focus only on a single risk factor, even when evidence strongly suggests multifactorial etiologies. For example, pilot studies demonstrate the ability to increase maternal knowledge about health literacy and preterm birth but show no proven impact on birth outcomes [47]. Specific interventions such as state Medicaid-sponsored enhanced prenatal care with targeted home visits by nurses can address multiple factors and may have more direct impact on outcomes [48]. Group prenatal care visits (centering) are showing early promise in not only improving birth outcomes but reducing the racial disparity in preterm birth for black and white women [49]. Other targets for potential intervention include food security, elimination of environmental hazards, smoking cessation, the promotion of breastfeeding, and undoing racism [50,51].

A growing number of multi-dimensional, place-based efforts to address racism are emerging and may further illuminate the paths by which communities may achieve maternal-child health equity [52]. For example, the “Best Babies Zone” being implemented in three urban neighborhoods utilizes “resident-driven strategies for decreasing the root causes of toxic stress and poor birth outcomes” by “aligning resources, building community leadership, and transforming educational opportunities, economic development, and community systems in concentrated neighborhoods” [53].

Additionally, further research is needed on formal and informal policies impacting health systems and community-level factors that improve care and support of women before, during, and after conception, as well as the broader upstream social and economic context and built environment in which people live [54].

### Limitations

This study is subject to all the known limitations of vital statistics data and, specifically, the designation of race and ethnicity on birth certificates. The lack of inclusion of paternal race-ethnicity is another relevant factor emphasized by Fulda et al [55]. Our study is also limited by the geographic constraints of using county-level data [56]. There is a clear tension between inclusion of more counties and the need to maintain confidentiality and to have sufficient births to achieve some stability of rates at the local-area level. In future work addressing the social context of birth outcomes, assessment of county LBW may be less useful in larger population counties. Analysis at a more granular level, i.e., census tract, without compromising stability, could gain more meaningful insights into neighborhood-level causes of convergence. Within counties or even cities, GIS techniques such as kernel density estimation can demonstrate hotspots in which more than 20% of births have either premature delivery or low birthweight, helping to focus programmatic interventions even more specifically than can aggregations of data at the county, neighborhood, or even census tract level [57]. Determining local area variation among outcomes, such as preterm birth, are also worthy of study. However, the authors chose to focus on birth weight given its consistent measurement during the study period. Further research will be needed to better understand why some communities are achieving greater progress toward lowering LBW rates and reducing the black-white gap.

## Conclusions

LBW is a significant driver of the black-white gap in birth outcomes and in the resultant impact on child health and development over the life course. It is thus an appropriate target for achieving greater racial equality in birth outcomes. Progress has been slow at the national level. Local-area variation, and especially the ability of some counties to make progress in reducing black LBW rates and the black-white disparity, suggests that there are paths for other counties to follow in achieving more optimal and equitable birth outcomes for everyone. Further research is needed to understand what is working in positive exemplar counties and to test multi-dimensional interventions for improving birth outcome equity in high-disparity locales.
